# Development of a tendon-driven serial manipulator for an aquatic autonomous surface vehicle

**DOI:** 10.3389/frobt.2026.1801347

**Published:** 2026-04-24

**Authors:** Alaa Khalifa, Keir Groves, Joaquin Carrasco

**Affiliations:** 1 School of Electrical and Electronic Engineering, The University of Manchester, Manchester, United Kingdom; 2 Department of Industrial Electronics and Control Engineering, Faculty of Electronic Engineering, Menoufia University, Menouf, Egypt

**Keywords:** extreme environment, kinematics, MallARD ASV platform, nuclear robotics, underwater robotics, wire-driven manipulator

## Abstract

This paper proposes a two-degree-of-freedom (2-DOF) tendon-driven manipulator to be attached to an aquatic Autonomous Surface Vehicle ASV (MallARD platform is an example). This attachment will expand the ASV’s reachable workspace. It also enables the ASV to perform underwater tasks as well as those performed on the water’s surface. The MallARD DOFs are invested in reducing the DOFs of the proposed manipulator. The actuators for the proposed manipulator are installed in the base, above the water line. Wires are used to transmit the power to the manipulator’s joints. The proposed wire-driven manipulator can work under high radiation, carry a large payload, and be easily isolated from water. The design of the manipulator is described in detail. The inverse kinematics closed-form solution has been derived analytically. A real-world version of the proposed wire-driven manipulator has been successfully manufactured and tested. The experimental setup is constructed utilizing the proposed manipulator. The experimental results display the feasibility of the proposed tendon-driven serial manipulator. They show that the RMS error between the desired and actual values in each joint is less than $2.1^{o}$ . Hence, the proposed wire-driven manipulator can be utilized for underwater applications.

## 1 Introduction

Autonomous surface vehicles (ASVs) are maritime vessels that can operate in different aquatic environments without a crew. ASVs are distinguished by their high mobility, low cost, and high levels of intelligence and autonomy (Peng et al., 2020). They give exceptional capabilities for carrying out diverse operations in hazardous maritime environments without endangering human life (Liu et al., 2016; Roberts, 2008). ASVs can be used for a wide range of marine applications such as marine surveying, search and rescue, environmental monitoring, offshore installation protection, and maritime security (Zereik et al., 2018; Elkins et al., 2010). Moreover, ASVs have been shown in military applications to play a significant role in numerous operations such as reconnaissance, surveillance, intelligence, and mine countermeasures (Melo and Matos, 2008). Generally, the employment of ASVs helps to minimise human costs, increase safety, and extend operations in hazardous environments.

Earlier ASVs were developed to operate in outdoor environments. However, there is currently a lack of ASVs that are suitable for an enclosed aquatic environment. In previous work, the authors describe an ASV [MallARD (Groves et al., 2019)] that made it possible to automate monitoring and inspection operations in wet silos and storage pools of nuclear fuel. The MallARD can be used to inspect and monitor various conditions on the surface of the Spent Fuel Pool (SFP) including radiation levels, temperature, and visual checks for degradation, cracks, corrosion, etc. This is especially important when legacy silos or pools are emptied for decommissioning and the pool walls’ decontamination must be validated. MallARD includes a localization technique that operates, without the need for external infrastructure, in restricted GPS-denied environments. In several experiments, the MallARD platform was proven to outperform the earlier ASVs (Groves et al., 2019). MallARD has the ability to operate on the water’s surface and features a wide flexible payload area that can hold a robotic manipulator outfitted with a variety of sensing devices.

It is worth mentioning that underwater Remotely Operated Vehicles (ROVs) are currently used for many tasks in SFPs (Dong et al., 2016, Dong et al., 2019). However, they have problems with tether management and snagging in addition to the need for decontaminating the tether after each mission. If ROVs are driven close to the bottom, they kick up sediment. This will make them blind and hard to see. They can not localise themselves either. So, if ASV can do any task with a long reach arm attached to its platform, this will be beneficial in many respects. Also, ROVs cannot currently give a good estimate of their location in a pond, but a surface vehicle with an arm can tell a good estimate of the end-effector pose.

The main motivation of the present paper is to propose a robotic manipulator to be attached to a small ASV platform that is suited to use in confined environments (MallARD as an example). ASV can only inspect and monitor on the surface - the arm would allow the ASV to do this underwater as well. The arm could hold a camera for inspection or a radiation detector. This robotic arm should be easy to implement and isolate from water. It should also be able to work under high radiation and carry a large payload.

A manipulator (robot arm) is believed to be the ideal instrument for performing underwater inspection monitoring and intervention procedures (Yuh and West, 2001; Antonelli, 2014; Sivčev et al., 2018). Hydraulic systems generally have greater payload capacity than electrical systems. For this reason, as well as since they have fewer components, hydraulic systems are often more compact than other systems with the same carrying capability. Because of these advantages, hydraulic oil drives the great majority of commercial underwater manipulators. Several research groups have designed and built prototype/experimental hydraulic underwater manipulators. Yao et al. (2009) and Zhang et al. (2014) introduced several manipulators with robot configurations and specifications like the commercially existing ones used on ROVs. Zuyao et al. (2011), on the other hand, concentrated on an unusual configuration that had three degrees of freedom. Despite their obvious advantages, hydraulically powered manipulators have several disadvantages. They offer low positional precision unlike their electrical counterparts. Hence, they are not suitable for precisely controlling the interaction force with the surroundings during contact operations (Terribile et al., 1993). Another disadvantage of hydraulic systems is the possibility of small hydraulic fluid leakage, which is difficult to resolve. Spilling oil into radiation-contaminated water is a real problem in the nuclear industry. Both drawbacks increase the need for high-quality materials and standards in component manufacturing, raising the cost of hydraulic systems. Furthermore, hydraulic manipulators necessitate the use of auxiliary equipment like a hydraulic pump, filters, valves, regulators, and so on.

Electrical-driven underwater manipulators are less common in commercial applications. A brushless DC motor, which has harmonic drive gears having a high reduction ratio and low backlash, is extensively used as an electric actuator. Lewandowski et al. (2008) developed a subsea manipulator enabling autonomous interventions and underwater retrieval. Hamed et al. (2014) designed a 4-DOF electrical underwater manipulator named Kavosh-4 to be used inside a towing tank. LAFMIA-UMI-I, a 3-DOF electrical underwater manipulator, was created to be installed on a tiny submarine (Cobos-Guzman et al., 2013). Xu et al. (2010) have designed a 3-DOF electrical-driven underwater robotic manipulator, which has been developed and tested latterly by Xiao et al. (2011) and Shen et al. (2011). Fernández et al. (2013) described a modification to a commercially existing electrical-driven manipulator known as ARM 5E as part of the RAUVI project. REACH ROBOTICS (2026) (formerly Blueprint Lab) recently introduced the Reach Alpha arm, which would be attached to ASV, but it is too short to be useful. They also presented the Reach Bravo arm, which is big enough but it is too heavy and expensive. Generally, the fundamental benefit of underwater electrically powered manipulators is their ability to perform accurate motion and torque/force control since they are intrinsically equal to industrial arms. However, they frequently fail to match the reliability, speed, and force or strength required for most intervention jobs (Hildebrandt et al., 2009). Moreover, it is hard to fulfill the waterproofing as the electrical actuators are located underwater. Also, it is difficult to use them in SFPs as all their electronics are near radiation. A comprehensive comparison of existing systems is already available in the literature. Specifically, the review paper by Sivčev et al. (2018), provides a detailed comparative table summarizing key specifications such as DOF, weight, power, and actuator configuration.

A wire-driven serial manipulator with actuators located in the base, which can be above the waterline, offers numerous benefits over a manipulator with actuators installed in the joints. The motors are kept out of the water to avoid sealing the main part of the arm and to keep electronics away from radiation. Because there are no heavy actuators installed in the joints of the wire-driven manipulator, it is feasible to lower the arm’s total weight, allowing actuators to be small as well as the arm to be slim. Furthermore, because the arm has no electronic components below the water, it can serve in extreme environments like underwater and under intense radiation. On the other hand, additional components such as pulleys and wires are needed for this type of manipulator. Also, the possibility of wire slacking may arise, and pre-tensioning is required. In this article, taking the above advantages, we aim to develop a wire-driven serial manipulator which can be attached to an ASV platform for underwater applications. Many wire-driven serial manipulators have been presented until now, but none have been designed specifically for underwater applications. One of the traditional approaches for building a redundant serial manipulator is to organise the wires radially and perform the bending movement by relaxing and pulling the wires (Hirose, 1981; Camarillo et al., 2009). A wire-driven long manipulator with a weight compensation mechanism has been developed for the decommissioning of the Fukushima Daiichi NPPs (Horigome et al., 2014, Endo et al., 2019). In this work, we will concentrate on a robotic manipulator with rigid links actuated by tendons and pulleys to construct a thinner arm than a traditional one.

Tendon-driven systems are indeed well established in fields such as medical robotics (e.g., surgical instruments). However, adapting such systems for aquatic environments, especially those involving simultaneous radiation exposure and underwater operation, introduces unique challenges that have not been addressed in existing literature. In medical applications, tendon-driven designs prioritize miniaturization and precision within sterile, controlled environments. In contrast, our design is tailored for an uncontrolled aquatic setting, with primary emphasis on radiation hardening, water isolation, and payload capacity. The testing framework by Salem et al. (2023) offers valuable practices for underwater system validation. The approach by Cornejo et al. (2025) ensures that the tendon transmission systems supporting modularity and scalability for integration with aquatic platforms.

The main objective of this work is the design of a tendon-driven manipulator to be attached to an ASV, our example is MallARD, for underwater applications. The reachable workspace of the ASV will be enlarged due to the attachment of the robotic arm. In addition, the ASV will be able to perform both surface and underwater tasks. We particularly concentrate herein on the wire-driven manipulator in which all actuators are installed on the base, above water. The technique of driving the manipulator joints by wires enables us to construct a lightweight arm architecture while obtaining a large payload capability, easy water isolation and strong radiation resistance. To the best of our knowledge, this is the first wire-driven serial manipulator designed specifically for underwater applications. The purpose of this paper is to design and build a 2-DOF wire-driven serial manipulator and validate its viability through simple motion experiments. Because MallARD is holonomic, we can take advantage of its DOFs to achieve a good range of motion even though the arm is only 2-DOF. For another vehicle, the arm may require more DOF, possibly a revolute joint at the base.

The following is how the reminder of this paper is organized. Section 2 describes the proposed wire-driven serial manipulator in detail. Kinematics analysis of the proposed wire-driven manipulator is presented in Section 3. Section 4 discusses the experimental arrangement and system validation. Experimental results are introduced in Section 5, while Section 6 summarises the conclusions and future work.

## 2 Description of the proposed tendon-driven manipulator

This new tendon-driven underwater manipulator is based on a serial manipulator that possesses two DOFs. Unlike serial manipulators that are directly actuated by electric motors, the proposed tendon-driven manipulator does not require bulky actuators to be attached to its joints. Instead, wires are used to transmit the motion to the robot joints from the actuators that are fixed on the robot base, above the water surface. Therefore, from a practical standpoint, the tendon-driven manipulator has the best payload capacity, water isolation, and ability to work under high radiation. Furthermore, it is easier to construct and less expensive than a manipulator that is actuated using hydraulic actuators.

**FIGURE 1:**
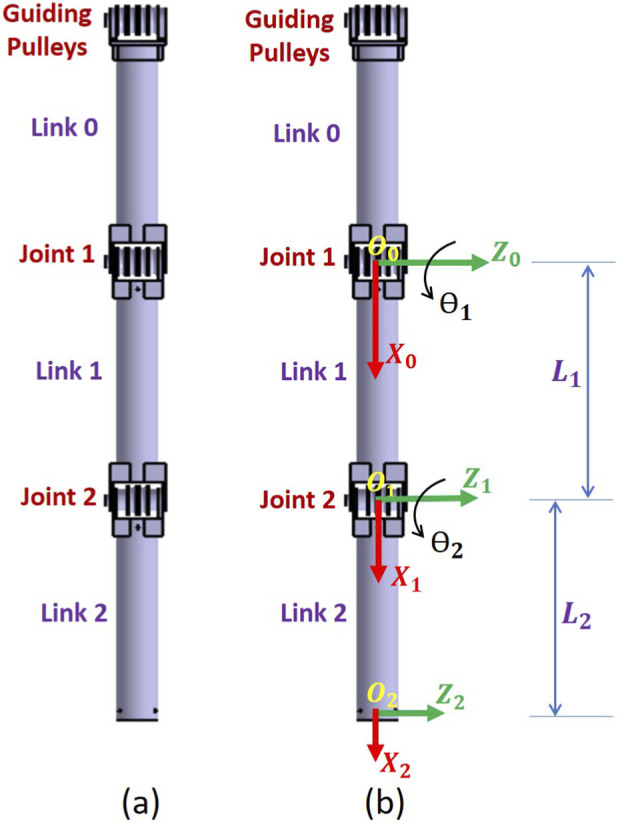
The proposed tendon-driven serial manipulator: **(a)** CAD model **(b)** Kinematic vector representation.

Figure 1a depicts the Computer-Aided Design (CAD) model for the proposed tendon-driven serial manipulator in its extended home position configuration. This proposed manipulator composes of a fixed base link, two movable links, and two revolute joints. For the proposed manipulator, the two axes of the two revolute joints are parallel to each other. The first revolute joint connects one end of the first link (Link 1) to the fixed base link (Link 0). While the second revolute joint connects the other end of the first link to one end of the second link (Link 2). The other end of the second link will be used for attaching any required sensor or end-effector. It is worth mentioning that the length of the fixed link (Link 0) may be modified according to the requirements of the task. All manipulator links take the shape of hollow cylinder to reduce the water resistance against the movement of these links while performing any task. Moreover, these hollow cylinders will allow the wires to pass through them easily.

The actuators (rotary electric motors) are placed outside the robot and the power is transmitted to the proposed manipulator by wires and free passive pulleys, which control the revolute joints’ motion. A flexible wire (flexible rope) bends smoothly and offers a flexible way of transmitting this power. The wire will be pulled or slackened by turning the motor. Four passive pulleys are free to rotate around the axis of the first revolute joint. While two passive pulleys are free to rotate around the axis of the second revolute joint. The guiding pulley base is fixed firmly to the upper tip of the fixed base link (Link 0). The axis of this guiding pulley base is parallel to the two revolute joints’ axes. It contains four passive pulleys which are free to rotate around its axis.

Four wires are used to transmit the motion from four rotary electric motors to the two joints of the proposed manipulator (two wires for each joint). Each wire is connected firmly from one end to a fixed pulley attached to a rotary motor shaft. The other end of each wire is connected firmly to the first tip of either Link 1 or Link 2 to actuate Joint 1 or Joint 2, respectively. The four wires are under tension all the time.

**FIGURE 2:**
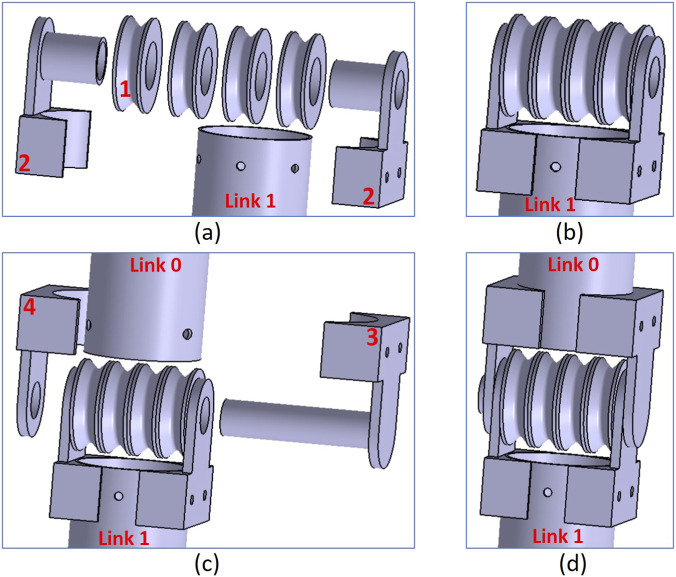
Assembly sequence of Joint 1 structure: **(a)** Aligning lower parts on Link 1 **(b)** Sub-assembly result **(c)** Aligning upper parts on Link 0 **(d)** Final assembly of Joint 1.

The assembly sequence for the structure of Joint 1 is shown in Figure 2. In Figure 2a, four hollow pulleys (like the part distinguished by number 1) are aligned to the hollow shafts of the two parts (indicated by number 2) and fixed around the tube of Link 1 using screws and nuts. Figure 2b presents the result of this sub-assembly. In Figure 2c, Joint 1 shaft (the part indicated by number 3) is passed inside the hollow shafts of the previous sub-assembly and fixed around the tube of Link 0 using screws and nuts. Also, part 4 is aligned to the shaft of part 3 and fixed around Link 0 using screws and nuts. The final result of this assembly is illustrated in Figure 2d. A small clearance between the shaft of part 3, part 2, and part 4 allows Link 1 and its fixed parts to rotate freely around Joint 1 shaft. Furthermore, a small clearance between part 1 and part 2 allows the four pulleys to rotate freely around their axis. It is worth mentioning that, in Figure 2, a hole on the upper end of Link 1 is made for fixing an end of a wire. This wire will be knotted around a screw which will be fixed to this hole by a nut. Pulling this wire causes Link 1 and its fixed parts to rotate in a specific direction relative to the fixed Link 0. Another hole in the opposite position on the same upper end of Link 1 is made for fixing an end of another wire by the same technique. Pulling this wire causes Link 1 to rotate in the opposite direction relative to the fixed Link 0.

Joint 2 is constructed in the same manner. The only difference is that Joint 2 contains only two passive free pulleys. Two hollow spacer parts are used to substitute the missing two pulleys. Moreover, in a similar manner, the guiding pulleys base is assembled. Because all driving tendons are routed through passive rotational pulleys of adequate diameter, the friction forces between the tendons and structural sections are considerably minimised due to the passive pulleys’ rolling contact.

**FIGURE 3:**
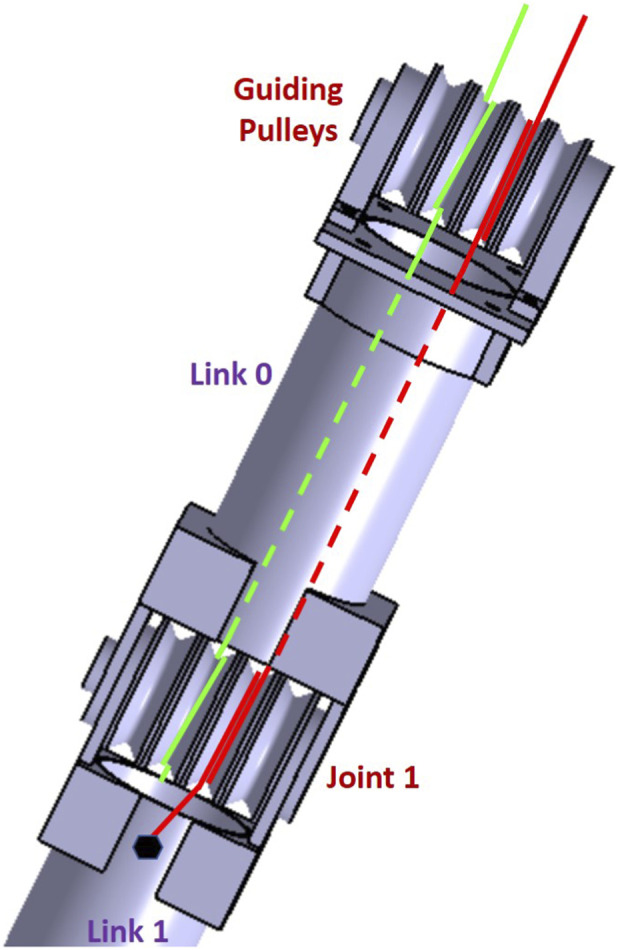
Joint 1 actuation using two wires.

To actuate the first manipulator’s joint, two wires pass through the guiding free pulleys, Joint 1 free pulleys, and attached to the first end of Link 1 in opposite locations as shown in Figure 3. Link 1 can rotate around the axis of Joint 1 by pulling one wire and relaxing the other. Similarly, to actuate the second manipulator’s joint, two wires pass through the guiding free pulleys, Joint 1 pulleys, Joint 2 pulleys, and are attached to the first end of Link 2 in opposite locations. This will allow Link 2 to rotate freely in different directions around the axis of Joint 2.

## 3 Kinematic analysis of the proposed tendon-driven manipulator

To utilize the underwater manipulator in any application, kinematics and control algorithms must be applied. There is a wealth of literature concerning manipulator kinematics that can be introduced to underwater robotics, some of them are available in Spong et al. (2006), Siciliano et al. (2009), Niku (2020).

### 3.1 Inverse kinematic analysis

Figure 1b displays the kinematic vector representation for the proposed tendon-driven serial manipulator in its extended home position configuration. Inverse kinematics is the procedure for determining the movement of the robot actuators to obtain the required end-effector pose. For the inverse kinematics of the proposed manipulator, the position vector P for a point $O_{2}$ placed at the centre of the end-effector location is provided as

$$P=\left[\begin{array}{c} p_{x}\\ p_{y}\\ 0 \end{array}\right].$$ (1)

While the requirement is to determine the angular movements of the four electric motors $\theta_{m}$ that indicated by

$$\theta_{m}=\left[\begin{array}{c} \theta_{m1}\\ \theta_{m2}\\ \theta_{m3}\\ \theta_{m4} \end{array}\right].$$ (2)

A fixed reference Cartesian frame $O_{0}$ - $X_{0}Y_{0}Z_{0}$ is affixed to the centre of the first joint (Joint 1), as shown in Figure 1b. The $Z_{0}$ -axis of the reference frame $O_{0}$ - $X_{0}Y_{0}Z_{0}$ is placed at the Joint 1 axis. $X_{0}$ -axis is perpendicular to the $Z_{0}$ -axis, points downward, and passes through Joint 1 centre. $Y_{0}$ -axis fulfils the right-hand rule. Also, a body frame $O_{1}$ - $X_{1}Y_{1}Z_{1}$ is affixed to the centre of Joint 2 and has an identical pose as the reference frame $O_{0}$ - $X_{0}Y_{0}Z_{0}$ . Similarly, a body frame $O_{2}$ - $X_{2}Y_{2}Z_{2}$ is affixed to the end-effector location and has an identical pose as the reference frame $O_{0}$ - $X_{0}Y_{0}Z_{0}$ .

The kinematics analysis can be performed in two basic steps. The first step is to derive a kinematic relationship between the end effector’s location and the open-loop chain’s joint angles. The Denavit-Hartenberg (DH) procedure described by Spong et al. (2006) can be used to accomplish this step. The second step is to derive a kinematic relationship between the angles of manipulator joints and the displacements of the wires and consequently the rotational displacements of the electric actuators.

Concerning the first step, the DH parameters for the proposed manipulator are illustrated in Table 1. $\theta_{i},a_{i},\alpha_{i}$ , and $d_{i}$ parameters are commonly denoting the joint angle, link length, link twist, and link offset, respectively. $L_{1}$ and $L_{2}$ are the lengths of Link 1 and Link 2, respectively. The length for both $L_{1}$ and $L_{2}$ is equal to 0.39 m. $\theta_{i}$ parameters are shown combined with * sign to indicate that these parameters are variables. Where i equals 1 or 2 for our 2-DOF manipulator. Using the kinematics algorithm, discussed in detail by Spong et al. (2006), we can easily conclude that

$$\cos\left(\theta_{2}\right)=\frac{p_{x}^{2}+p_{y}^{2}-L_{1}^{2}-L_{2}^{2}}{2L_{1}L_{2}},$$ (3)

$$\sin\left(\theta_{2}\right)=-\sqrt{1-\cos^{2}\left(\theta_{2}\right)},$$ (4)

$$\theta_{2}=\tan^{-1}\left(\frac{\sin\left(\theta_{2}\right)}{\cos\left(\theta_{2}\right)}\right),$$ (5)

$$\theta_{1}=\tan^{-1}\left(\frac{p_{y}}{p_{x}}\right)-\tan^{-1}\left(\frac{L_{2}\sin\left(\theta_{2}\right)}{L_{1}+L_{2}\cos\left(\theta_{2}\right)}\right).$$ (6)

**TABLE 1:** Link parameters for the proposed 2-DOF manipulator.

Link	$\theta_{i}$	$a_{i}$	$\alpha_{i}$	$d_{i}$
1	${\theta *}_{1}$	$L_{1}$	0	0
2	${\theta *}_{2}$	$L_{2}$	0	0

So, given the position vector P and using Equations 3–6, we can easily construct the joint angles two-dimensional vector $\theta ={\left[\theta_{1}\;\theta_{2}\right]}^{T}$ .

Concerning the second step, mathematically, a linear transformation relates the angles of the manipulator’s joints and wire displacements. By inspecting the wire routing topology, the transformation matrix can be acquired utilizing the transmission lines concept (Tsai, 1999). The transformation matrix is generally a function of wire routing topology, pulley size, and manipulator posture. Though, the matrix was proven to be independent of manipulator posture in the case of serial manipulators where wires are routed sequentially from a joint to another (Tsai, 1999).

The equations can be combined into a matrix as

ζ=Rθ, (7)

where $\zeta ={\left[\zeta_{1}\;\zeta_{2}\;\zeta_{3}\;\zeta_{4}\right]}^{T}$ represents a four-dimensional vector of wire displacements, $\theta ={\left[\theta_{1}\;\theta_{2}\right]}^{T}$ indicates a two-dimensional vector for the angles of manipulator’s joints, and R is a four-by-two transformation matrix. The elements of R are independent of manipulator posture and are functions of wire routing topology and pulley sizes. The R matrix for the proposed manipulator is

$$R=r\left[\begin{array}{cc} -1 & 0\\ 1 & 0\\ -1 & -1\\ 1 & 1 \end{array}\right],$$ (8)

where r is the radius of the pulley. For simplicity, we have chosen all the pulleys in the proposed manipulator with the same radius r . Finally, to convert the tendon displacements ζ into angular displacements of the electric motors $\theta_{m}$ , we can use

$$\theta_{m}=\frac{1}{r}\zeta .$$ (9)

### 3.2 Jacobian and singularity analysis

The Jacobian matrix for the 2 DOF manipulator, which relates joint velocities to end effector velocities, has been derived and is given by:

$$J\left(\theta_{1},\theta_{2}\right)=\left[\begin{array}{cc} -L_{1}\sin\left(\theta_{1}\right)-L_{2}\sin\left(\theta_{1}+\theta_{2}\right) & -L_{2}\sin\left(\theta_{1}+\theta_{2}\right)\\ L_{1}\cos\left(\theta_{1}\right)+L_{2}\cos\left(\theta_{1}+\theta_{2}\right) & L_{2}\cos\left(\theta_{1}+\theta_{2}\right) \end{array}\right].$$ (10)

The determinant of the Jacobian is: $\det\left(J\right)=L_{1}L_{2}\sin\left(\theta_{2}\right)$ . Singularities occur when det(J)=0 , which happens when:

-
$\theta_{2}=0{}^{\circ}$ (arm fully extended). This configuration can be avoided in the control algorithm and the manipulator’s motion planner, as discussed later in Section 4.3.
-
$\theta_{2}=\pm 180{}^{\circ}$ (arm fully folded back). This configuration will not be reached, as $\theta_{2}$ is limited by the manipulator architecture to ±90° .

The manipulability index is given by:

$$w=\sqrt{\det\left(JJ^{⊤}\right)}=|L_{1}L_{2}\sin\left(\theta_{2}\right)|.$$ (11)

This index reaches its maximum when $|\sin\left(\theta_{2}\right)|=1$ , i.e., $\theta_{2}=\pm 90{}^{\circ}$ .

## 4 Experimental arrangement and system validation

**FIGURE 4:**
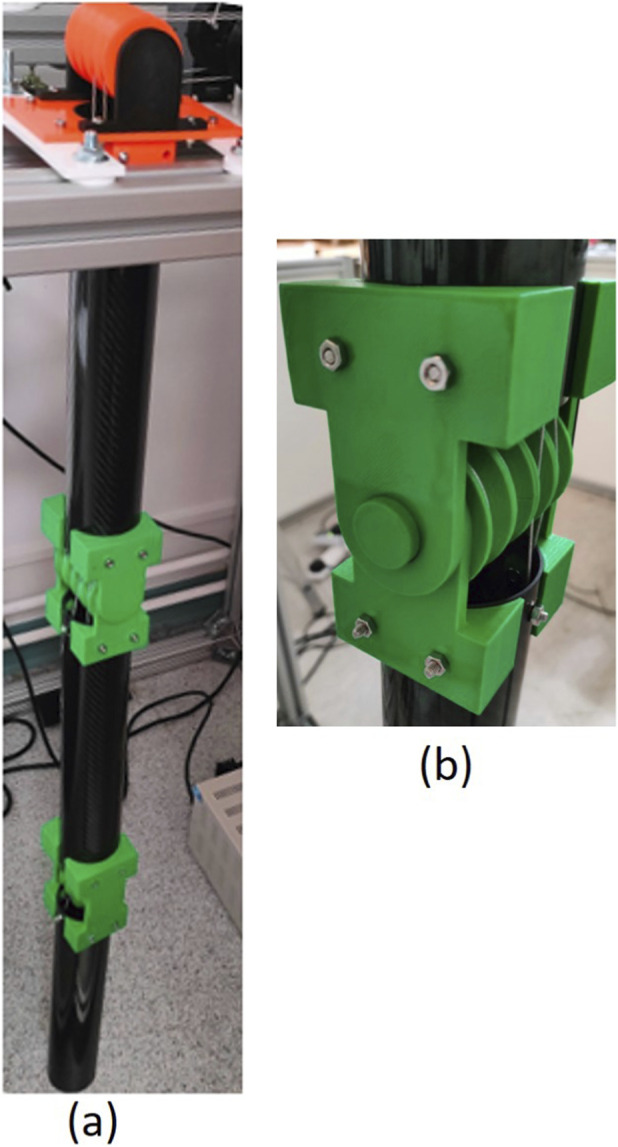
A real 2-DOF wire-driven serial manipulator: **(a)** the entire manipulator **(b)** Joint 1.

**FIGURE 5:**
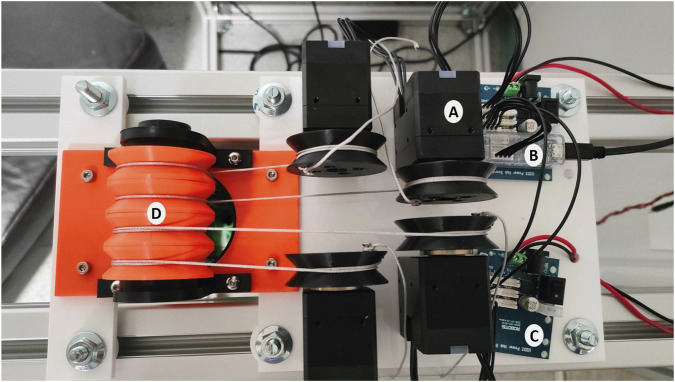
Experimental setup (Top view).

A real 2-DOF wire-driven manipulator, as shown in Figure 4a, was manufactured to validate the proposed robot design and its motion capability. Figure 4b depicts an enlarged view of the real construction of Joint 1. A hollow cylinder (tube) from high strength carbon fibre was cut to form the manipulator’s links (Links 0, 1, 2). The incorporated parts in the guiding pulleys base, Joint 1, and Joint 2 have been manufactured using 3D printing. In real and commercial robot, the material for these parts may be stainless steel or PEEK (Ensinger, 2026). A discussion on how cost effective materials affect joint reliability is presented in Salem et al. (2025). Figure 5 illustrates a top view of the experimental setup where four motors are utilized to control the four wires and consequently the two joints of the proposed manipulator. The following subsections present the specifics of the hardware, software, and employed motion planner.

### 4.1 Hardware implementation

The following tools are employed to construct the experimental setup.

#### 4.1.1 DC servo motors (Robotis - DYNAMIXEL XH540-W270-R)

DYNAMIXEL is a manipulator-exclusive smart actuator that incorporates a DC motor, reduction gearhead, driver, controller and network into a single DC servo module. It has a small encoder with high resolution that can accomplish ultra-high positioning. For our proposed system, the Robotis-DYNAMIXEL XH540-W270-R module is used. The full specifications of this motor module can be found in ROBOTIS e-Manual (2026c). Four motors are employed to control the four tendons and hence the two joints of the proposed tendon-driven manipulator.

#### 4.1.2 ROBOTIS U2D2

U2D2 is a mini-USB communication converter that allows to operate and control the DYNAMIXEL motors from the PC. It connects to the PC via a USB cable and protects the USB terminals from damage. It has embedded communication connectors for easier access and control of the DYNAMIXEL X series. It serves as an interface between the proposed manipulator and the PC, receiving sensors’ data and sending commands to the manipulator’s actuators based on the control algorithm. As U2D2 does not deliver power to the DYNAMIXEL motor, an external power supply is required. The full specifications of ROBOTIS U2D2 can be found in ROBOTIS e-Manual (2026a).

#### 4.1.3 ROBOTIS - U2D2 PHB SET

This module has been developed specially as a driver for the employed DYNAMIXEL motor. The U2D2 Power Hub Board (PHB) works in tandem with the U2D2 in order to provide DYNAMIXELs with several external power sources. Two U2D2 PHB SET units are used to drive the four DYNAMIXEL motors; One unit for driving two motors. This is due to the current limitation of the board. The full specifications of ROBOTIS U2D2 PHB SET can be found in ROBOTIS e-Manual (2026b).

#### 4.1.4 The manufactured tendon-driven manipulator

This is the proposed 2-DOF tendon-driven manipulator to be attached to the aquatic autonomous surface vehicle (MallARD is our example). Section 2 contains a comprehensive description of this proposed manipulator.

#### 4.1.5 Power supply unit

It provides DC 12 V to the four DYNAMIXEL motors via the two U2D2 PHB SET.

#### 4.1.6 Personal computer (PC)

It is the controller console which is programmed to solve the inverse kinematics of the proposed tendon-driven manipulator and control the movement of the four rotary electric motors simultaneously according to the trajectory planning.

**FIGURE 6:**
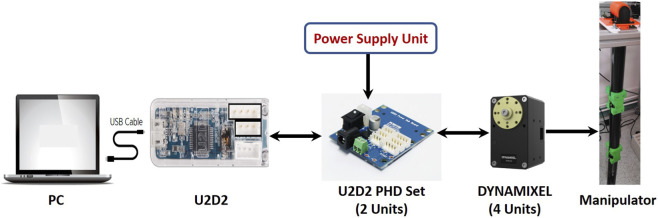
Block diagram of the experimental system.

The system block diagram, presented in Figure 6, demonstrates the arrangement of the system’s components and the signal flow between them. In Figure 6, the PC is connected to the U2D2 unit via a USB cable. Serial communication is utilized to connect the U2D2 unit with the U2D2 PHB SET units and the four servo motors.

### 4.2 Designed software program

Software is required to accomplish the hardware interface and gather, display, analyse, and store measurements. Robot Operating System (ROS) is a common framework for controlling many robots nowadays. ROS is essentially a framework for communicating between many programs or processes (Joseph, 2018).

ROS Melodic under Ubuntu 18.04.5 LTS operating system (with both Python and C++ nodes) is used to connect the proposed tendon-driven manipulator to the PC. Initially, in real-time, a sample node was created for commanding the four actuators of the two joints simultaneously and monitoring the present position. The operator determines the required end-effector position. A node converts this required position using inverse kinematics to commands which will be sent to the actuators. This node will publish the actuators commands in a specific topic with a certain rate. The other node will subscribe to this specific topic to get the desired values for the actuators and control their motions simultaneously. This, in turn, locates the proposed manipulator’s end-effector at the instructed position. As long as the system is operating, this process will continue. ROS bag is used to record all the topics data during the practical process.

**FIGURE 7:**
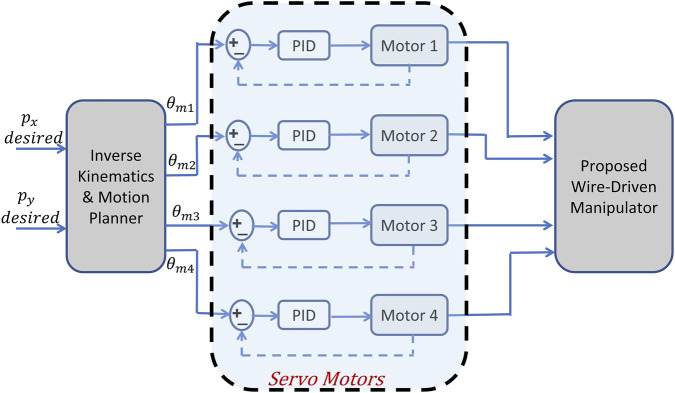
Schematic diagram of the control algorithm.

Figure 7 shows the schematic diagram of the control algorithm with the desired path as the reference input. PID controllers onboard the servo motors are used for controlling the actuators’ movements.

### 4.3 Manipulator’s motion planner

**FIGURE 8:**
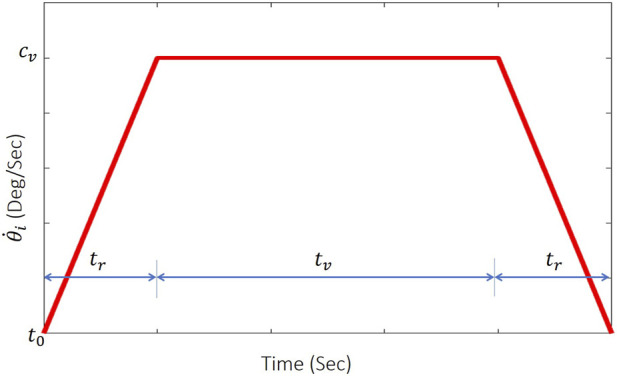
Velocity ramping function for the motion planner.

To avoid sudden movements and minimize force spikes caused by sudden velocity changes, a motion planner is used. It plans each joint trajectory and makes the manipulator move smoothly to the desired position. The planner’s role is to compute the appropriate angular positions and velocities, as a function of time, needed for the manipulator’s joints to move between two waypoints. The motion planner ramps each joint velocity from zero with a fixed acceleration $c_{a}$ to the highest velocity $c_{v}$ , then back down to zero upon reaching the goal angle value. Here, the target parameter is $\theta_{i}$ where i equals 1 or 2 for Joint 1 or Joint 2 angle value, respectively. Referring to Figure 8, when a new goal angle $\theta_{ig}$ and starting angle $\theta_{is}$ are sent to the manipulator’s motion planner, the current time is saved and set as $t_{0}$ . Then, $t_{r}$ and $t_{v}$ are computed as

$$t_{r}=\frac{c_{v}}{c_{a}},$$ (12)

$$t_{v}=\frac{\Delta \theta_{i}-t_{r}c_{v}}{c_{v}},$$ (13)

where $\Delta \theta_{i}=\theta_{ig}-\theta_{is}$ . Finally, the required joint angular position $\theta_{i}\left(t\right)$ and velocity ${\dot{\theta }}_{i}\left(t\right)$ are computed for each time step. ${\dot{\theta }}_{i}\left(t\right)$ is obtained directly from the function shown in Figure 8 and the value of $\theta_{i}\left(t\right)$ is determined by integrating the same function over the period from $t_{0}$ to the current time t .

## 5 Experimental results

An experiment has been performed as displayed in the video uploaded at the link^1^. This video demonstrates a practical implementation of the entire system in which the proposed tendon-driven serial manipulator is utilized. Furthermore, this video shows the capability of the proposed manipulator to move and reach different positions.

VICON motion capture system, which is a room with twelve cameras positioned around its walls, has been used for tracking the proposed manipulator motions and its joints’ angles values, $\theta_{1}$ and $\theta_{2}$ . To verify the accuracy of the entire system, the VICON measurement is employed as a ground truth. In typical testing, the VICON system’s accuracy was demonstrated to be sub-millimetre accurate (Merriaux et al., 2017), and this was confirmed by the system’s internal error calculation metric.

**FIGURE 9:**
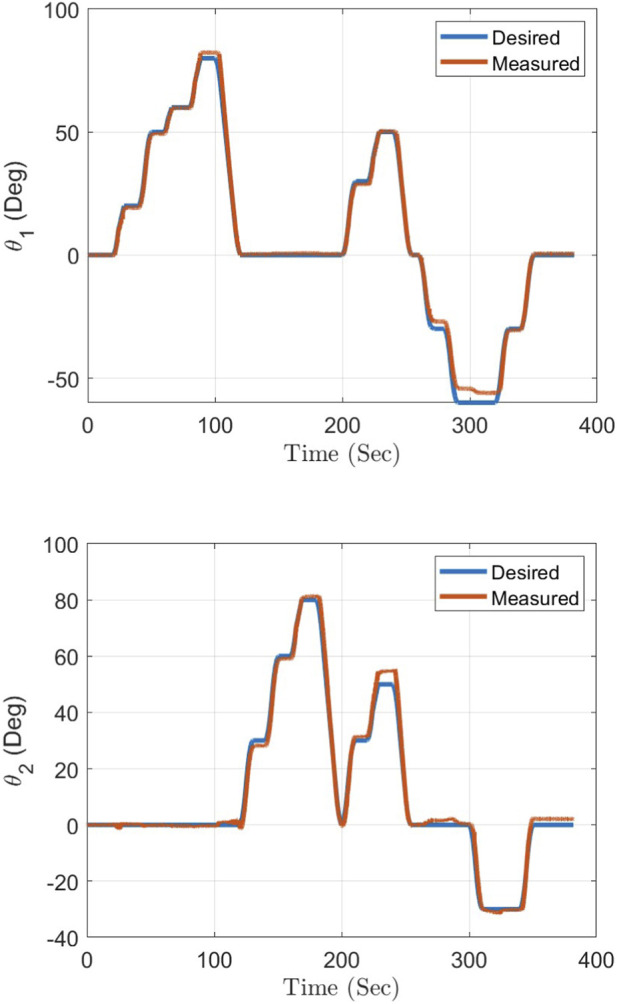
Experimental response of the proposed manipulator’s joints.

Three VICON markers were placed at Joint 1, Joint 2, and the end-effector location. The VICON system captures the coordinates of these markers throughout the whole experiment. Figure 9 shows the experimental response of the proposed manipulator’s joints as a result of moving the manipulator to different required positions. The measured values in this figure are obtained from the VICON system, while the desired values are acquired from the inverse kinematics solution. As can be seen in Figure 9, there is a tight match between the desired manipulator angles and the measured values from the VICON system. Over the experiment duration, the overall RMS error between the desired angle value and the measured one from the VICON system is $2.1^{o}$ and $1.9^{o}$ for $\theta_{1}$ and $\theta_{2}$ , respectively. These results display the feasibility of the proposed tendon-driven serial manipulator. Besides, they demonstrate that the control algorithm can track a trajectory.

## 6 Conclusion

This research presents a 2-DOF tendon-driven serial manipulator, with actuators located in the base above the water surface, to be mounted to an ASV. We take the MallARD platform as an example of the ASV. Attaching the proposed manipulator to the ASV enlarges its reachable workspace and allows it to perform both underwater and surface tasks. We use MallARD DOFs to reduce the DOFs of the proposed manipulator. The proposed robotic manipulator has a simple design, easiness of isolation from water, the ability to work under high radiation, and the ability to carry a large payload. The description of the manipulator design has been introduced in detail. The inverse kinematics closed-form solution has been acquired analytically using a two-step approach. The proposed wire-driven manipulator has been manufactured and tested successfully. Using this manufactured real model, the whole experimental setup has been accomplished. The movement of each actuator has been controlled using the PID controller onboard the servo motor. Motion planner has been used to make the manipulator movement goes smoothly. The obtained experimental results have proven the feasibility of the proposed tendon-driven manipulator and the efficiency of the employed algorithm to achieve good trajectory tracking. They illustrate that the RMS error in each joint angle value is less than $2.1^{o}$ . Therefore, the proposed tendon-driven serial manipulator can be attached to an ASV platform and applied efficiently for underwater applications.

A gripper will be fitted to the proposed manipulator for future research investigation. We intend to incorporate a physical tension management system (such as spring loaded tensioners or active antagonistic motor control) to maintain cable tension throughout operation. Moreover, the dynamics of the proposed manipulator and its effect on the ASV will be studied in detail. The comprehensive dynamic model will incorporate hydrodynamic drag, buoyancy, friction, Capstan effects, and cable elasticity to enable more accurate control and performance prediction in aquatic environments. Also, more experiments will be carried out underwater to assess the system’s performance and gather feedback. Finally, incorporating fault detection mechanisms would enhance system reliability and safety, particularly for deployment in critical environments such as nuclear facilities.

## Data Availability

The original contributions presented in the study are included in the article/supplementary material, further inquiries can be directed to the corresponding author.

## Footnotes

1
https://tinyurl.com/2p9rft8h

## References

[B1] Antonelli G. (2014). Underwater robots, 96. Springer.

[B2] Camarillo D. B. Carlson C. R. Salisbury J. K. (2009). Configuration tracking for continuum manipulators with coupled tendon drive. IEEE Transactions Robotics 25, 798–808. 10.1109/tro.2009.2022426

[B3] Cobos-Guzman S. Torres J. Lozano R. (2013). Design of an underwater robot manipulator for a telerobotic system. Robotica 31, 945–953. 10.1017/s0263574713000234

[B4] Cornejo J. Weitzenfeld A. Baca J. García Cena C. E. (2025). Aerospace bionic robotics: Beam-d technical standard of biomimetic engineering design methodology applied to mechatronics systems. Biomimetics 10, 668. 10.3390/biomimetics10100668 41149198 PMC12561782

[B5] Dong M. Chou W. Fang B. Yao G. Liu Q. (2016). Implementation of remotely operated vehicle for direct inspection of reactor pressure vessel and other water-filled infrastructure. J. Nucl. Sci. Technol. 53, 1086–1096. 10.1080/00223131.2015.1091395

[B6] Dong M. Li J. Chou W. (2019). A new positioning method for remotely operated vehicle of the nuclear power plant. Industrial Robot The International Journal Robotics Research Application 47, 177–186. 10.1108/ir-07-2019-0140

[B7] Elkins L. Sellers D. Monach W. R. (2010). The autonomous maritime navigation (amn) project: field tests, autonomous and cooperative behaviors, data fusion, sensors, and vehicles. J. Field Robotics 27, 790–818. 10.1002/rob.20367

[B8] Endo G. Horigome A. Takata A. (2019). Super dragon: a 10-m-long-coupled tendon-driven articulated manipulator. IEEE Robotics Automation Lett. 4, 934–941. 10.1109/lra.2019.2894855

[B9] Ensinger (2026). TECAPEEK ensinger plastics. Available online at: https://www.ensingerplastics.com/en-gb/shapes/high-performance-plastics/peek (Accessed February 1, 2026).

[B10] Fernández J. J. Prats M. Sanz P. J. García J. C. Marin R. Robinson M. (2013). Grasping for the seabed: developing a new underwater robot arm for shallow-water intervention. IEEE Robotics and Automation Mag. 20, 121–130. 10.1109/mra.2013.2248307

[B11] Groves K. West A. Gornicki K. Watson S. Carrasco J. Lennox B. (2019). Mallard: an autonomous aquatic surface vehicle for inspection and monitoring of wet nuclear storage facilities. Robotics 8, 47. 10.3390/robotics8020047

[B12] Hamed S. Mostashfi A. Madhkhan M. Esmaelean M. (2014). Development of a four-dof laboratory underwater manipulator for using in towing tank. Indian J. Sci. Res. 2, 895–899.

[B13] Hildebrandt M. Kerdels J. Albiez J. Kirchner F. (2009). “A multi-layered controller approach for high precision end-effector control of hydraulic underwater manipulator systems,” in OCEANS 2009 IEEE, 1–5.

[B14] Hirose S. (1981). Tensor actuated elastic manipulator. Proc. 6th IFToMM World Cong. 2, 978–981.

[B15] Horigome A. Yamada H. Endo G. Sen S. Hirose S. Fukushima E. F. (2014). “Development of a coupled tendon-driven 3d multi-joint manipulator,” in 2014 IEEE International conference on robotics and automation (ICRA) (IEEE), 5915–5920.

[B16] Joseph L. (2018). Robot operating system (ros) for absolute beginners. Springer.

[B17] Lewandowski C. Akin D. Dillow B. Limparis N. Carignan C. Singh H. (2008). “Development of a deep-sea robotic manipulator for autonomous sampling and retrieval,” in 2008 IEEE/OES Autonomous Underwater Vehicles (IEEE), 1–6.

[B18] Liu Z. Zhang Y. Yu X. Yuan C. (2016). Unmanned surface vehicles: an overview of developments and challenges. Annu. Rev. Control 41, 71–93. 10.1016/j.arcontrol.2016.04.018

[B19] Melo J. Matos A. (2008). “Guidance and control of an asv in auv tracking operations,” in OCEANS 2008 (IEEE), 1–7.

[B20] Merriaux P. Dupuis Y. Boutteau R. Vasseur P. Savatier X. (2017). A study of vicon system positioning performance. Sensors 17, 1591. 10.3390/s17071591 28686213 PMC5551098

[B21] Niku S. B. (2020). Introduction to robotics: analysis, control, applications. John Wiley and Sons.

[B22] Peng Z. Wang J. Wang D. Han Q.-L. (2020). An overview of recent advances in coordinated control of multiple autonomous surface vehicles. IEEE Trans. Industrial Inf. 17, 732–745. 10.1109/tii.2020.3004343

[B23] REACH ROBOTICS (2026). Advanced ROV manipulators. Available online at: https://reachrobotics.com/products/(Accessed February 1, 2026).

[B24] Roberts G. (2008). Trends in marine control systems. Annu. Reviews Control 32, 263–269. 10.1016/j.arcontrol.2008.08.002

[B25] ROBOTIS e-Manual (2026a). U2D2 emanual robotis. Available online at: https://emanual.robotis.com/docs/en/parts/interface/u2d2/(Accessed February 1, 2026).

[B26] ROBOTIS e-Manual (2026b). u2d2_power_hub emanual robotis. Available online at: https://emanual.robotis.com/docs/en/parts/interface/u2d2_power_hub/(Accessed February 1, 2026).

[B27] ROBOTIS e-Manual (2026c). XH540-W270-T/R emanual robotis. Available online at: https://emanual.robotis.com/docs/en/dxl/x/xh540-w270/(Accessed February 1, 2026).

[B28] Salem K. M. Rady M. Aly H. Elshimy H. (2023). Design and implementation of a six-degrees-of-freedom underwater remotely operated vehicle. Appl. Sci. 13, 6870. 10.3390/app13126870

[B29] Salem K. M. Mohamed M. S. ElMessmary M. H. Ehsan A. Elgharib A. ElShimy H. (2025). Design and development of cost-effective humanoid robots for enhanced human–robot interaction. Automation 6, 41. 10.3390/automation6030041

[B30] Shen X. Xu G. Yu K. Tang G. Xu X. (2011). “Development of a deep ocean master-slave electric manipulator control system,” in International Conference on Intelligent Robotics and Applications (Springer), 412–419.

[B31] Siciliano B. Sciavicco L. Villani L. Oriollo G. (2009). Robotics: modeling, planning and control. London: Springer.

[B32] Sivčev S. Coleman J. Omerdić E. Dooly G. Toal D. (2018). Underwater manipulators: a review. Ocean. Engineering 163, 431–450. 10.1016/j.oceaneng.2018.06.018

[B33] Spong M. W. Hutchinson S. Vidyasagar M. (2006). Robot modeling and control. 3. New York: Wiley.

[B34] Terribile A. Lanza R. Campaci R. (1993). “An innovative electromechanical underwater telemanipulator for underwater/nuclear and terrestrial applications,” in Regional meeting: nuclear energy in central Europe present and perspectives.

[B35] Tsai L.-W. (1999). Robot analysis: the mechanics of serial and parallel manipulators. John Wiley and Sons.

[B36] Xiao Z.-h. Xu G.-h. Peng F.-y. Tang G.-y. Shen X. Yang B. (2011). Development of a deep ocean electric autonomous manipulator. China Ocean. Eng. 25, 159–168. 10.1007/s13344-011-0014-9

[B37] Xu G. Xiao Z. Tang G. Shen X. Yang B. (2010). “A new deep ocean electrical driven manipulator: working system,” in The Twentieth International Offshore and Polar Engineering Conference (OnePetro).

[B38] Yao J. Wang L. Jia P. Wang Z. (2009). “Development of a 7-function hydraulic underwater manipulator system,” in 2009 International Conference on Mechatronics and Automation (IEEE), 1202–1206.

[B39] Yuh J. West M. (2001). Underwater robotics. Adv. Robot. 15, 609–639. 10.1163/156855301317033595

[B40] Zereik E. Bibuli M. Mišković N. Ridao P. Pascoal A. (2018). Challenges and future trends in marine robotics. Annu. Rev. Control 46, 350–368. 10.1016/j.arcontrol.2018.10.002

[B41] Zhang Q. Chen J. Huo L. Kong F. Du L. Cui S. (2014). “7000m pressure experiment of a deep-sea hydraulic manipulator system,” in 2014 Oceans-St. John’s (IEEE), 1–5.

[B42] Zuyao Y. Jie L. Weijia L. Zhongxiang X. Jinbo W. (2011). “Design and analysis of a three-dof underwater manipulator,” in Proceedings of 2011 International Conference on Fluid Power and Mechatronics (IEEE), 237–241.

